# Revealing Prdx4 as a potential diagnostic and therapeutic target for acute pancreatitis based on machine learning analysis

**DOI:** 10.1186/s12920-024-01854-2

**Published:** 2024-04-19

**Authors:** Zhonghua Lu, Yan Tang, Ruxue Qin, Ziyu Han, Hu Chen, Lijun Cao, Pinjie Zhang, Xiang Yang, Weili Yu, Na Cheng, Yun Sun

**Affiliations:** 1 The First Department of Critical Care Medicine, The Second Affiliated Hospital of Anhui Medical University, 678 Furong Road, 230601 Hefei, Anhui Province China; 2 Department of Rehabilitation Medicine, The Second Affiliated Hospital of Anhui Medical University, 678 Furong Road, 230601 Hefei, Anhui Province China; 3 School of Biomedical Engineering, Anhui Medical University, 81 Meishan Road, 230032 Hefei, Anhui Province China

**Keywords:** Acute pancreatitis (AP), Immune cell infiltration, Diagnostic value, Bioinformatics analysis, Machine learning

## Abstract

Acute pancreatitis (AP) is a common systemic inflammatory disease resulting from the activation of trypsinogen by various incentives in ICU. The annual incidence rate is approximately 30 out of 100,000. Some patients may progress to severe acute pancreatitis, with a mortality rate of up to 40%. Therefore, the goal of this article is to explore the key genes for effective diagnosis and treatment of AP. The analysis data for this study were merged from two GEO datasets. 1357 DEGs were used for functional enrichment and cMAP analysis, aiming to reveal the pathogenic genes and potential mechanisms of AP, as well as potential drugs for treating AP. Importantly, the study used LASSO and SVM-RFE machine learning to screen the most likely AP occurrence biomarker for Prdx4 among numerous candidate genes. A receiver operating characteristic of Prdx4 was used to estimate the incidence of AP. The ssGSEA algorithm was employed to investigate immune cell infiltration in AP. The biomarker Prdx4 gene exhibited significant associations with a majority of immune cells and was identified as being expressed in NKT cells, macrophages, granulocytes, and B cells based on single-cell transcriptome data. Finally, we found an increase in Prdx4 expression in the pancreatic tissue of AP mice through immunohistochemistry. After treatment with recombinant Prdx4, the pathological damage to the pancreatic tissue of AP mice was relieved. In conclusion, our study identified Prdx4 as a potential AP hub gene, providing a new target for treatment.

**Supplementary Information:**

The online version contains supplementary material available at 10.1186/s12920-024-01854-2.

## Background

Acute pancreatitis (AP) is a condition characterized by inflammation of the pancreas due to the excessive activation of pancreatic enzymes, often triggered by factors such as gallstones, high fat intake, and alcohol consumption. It not only causes damage to the pancreas itself, but also typically accompanies functional failure of other organs such as the liver, lungs, kidneys, and heart [1]. It is estimated that 33.74 cases of AP occur per 100,000 people annually, and severe AP is associated with a mortality rate of 10-30% [2]. Despite the various treatments currently utilized for acute pancreatitis, such as analgesia, fluid resuscitation, organ support, and nutrition [3], they have not effectively halted the pathogenesis of the condition. Furthermore, these treatments have shown limitations in enhancing the overall survival rate of patients. Therefore, to explore the key genes in the occurrence of acute pancreatitis, to further clarify its possible pathogenesis, may provide hope for the prevention and treatment of AP.

Over the past few decades, researchers have found that S100A6, S100A9, and S100A12 can predict severe pancreatitis in single-cell RNA sequencing experiments [4]. Bourgault J et al. identified blood proteins associated with AP based on genome-wide association meta-analysis and proteomic Mendelian randomization (MR) studies [5]. In chylomicron syndrome (CS), the gene expression variances between non-associated and CS-associated aps indicated reduced levels of miRNA, such as hsa-miR-21, hsa-miR-146a, and hsa-miR-106a, in non-CS-associated aps. The expression patterns and regulatory activities of non-CS associated AP and CS associated AP are largely opposite [6]. The genes are seen as the primary indicators for diagnosing AP.

In addition to inducing related genetic changes, the body’s dysfunctional response to inflammation in the pancreas is also linked to its immune system [7]. When AP occurred, macrophage numbers increased significantly, and macrophage interactions and intensity were significantly higher than in the control group, accompanied by abnormal regulation of the activity of signaling pathways, such as apoptosis, oxidative stress, lysosome, autophagy, iron death, and inflammation [7]. In addition, studies have found that there are different gene expression patterns and low neutrophil infiltration in AP related to CS [6]. Thus, the immune microenvironment serves as a critical link between key genes and AP.

Recently, bioinformation analysis and machine learning have become increasingly promising strategies for comprehensive and in-depth analysis of big data, such as transcriptome sequences, and interdisciplinary fields have permeated to develop more therapeutic approaches in clinical practice [8]. An analysis of weighted gene co-expression networks and least absolute shrinkage and selection operator regression analysis (LASSO) were performed to identify genes related to immune cell death in SAP [9]. A recursive feature elimination method, called support vector machine recursive feature elimination (SVM-RFE), has been found to be effective for the differentiation of autoimmune pancreatitis and pancreatic ductal adenocarcinoma in 18 F-FDG PET/CT [10]. In order to explore the genetic markers of AP occurrence and their role in the occurrence of the disease, it is undoubtedly the most important to clarify the key genes of AP based on machine learning and search for therapeutic drugs targeting the key genes or pathways.

In this study, we first analyzed the differential expression of pancreatic tissue genes between the control group and the AP group, identified the genes that might be related to the occurrence of AP, and combined LASSO regression and SVM-RFE algorithm to extract potential gene Prdx4 related to AP generation, and validated it on another dataset. We used ssGSEA (single sample gene set enrichment analysis) to compare immune cell populations in normal and AP samples and evaluate their association with potential genetic biomarkers for AP. As a whole, we uncovered a hitherto unknown gene that has the potential to guide future clinical treatment of acute pancreatitis patients.

## Materials and methods

### Data collection and download

We downloaded the GEO database (https://www.ncbi.nlm.nih.gov/geo/) for acute pancreatitis gene expression datasets containing GSE65146, GSE109227, and GSE3644. The GSE65146 dataset, consisting of 5 Control Samples and 6 samples of acute pancreatitis, was obtained from the GPL6246 platform of the Affymetrix Mouse Gene 1.0 ST Array. The GSE109227 dataset, consisting of 5 Control Samples and 6 acute pancreatitis samples, was obtained from the GPL6246 platform of [MoGene-1_0-st] Affymetrix Mouse Gene 1.0 ST Array. The GSE3644 dataset (6 Control Samples and 6 acute pancreatitis samples) was derived from the GPL339 platform of [MOE430A] Affymetrix Mouse Expression 430 A Array.

### Differentially expressed genes (DEGs) analysis

Background correction, normalization and gene symbol conversion were performed on the acute pancreatitis datasets (GSE65146 and GSE109227). We merged datasets using R software packages ‘limma’ and ‘sva’, removed batch effects using the Combat function, adjusted data using the ‘FDR’ method as the training set, and used the GSE3644 was as the validation dataset to confirm the analysis results. DEGs in the acute pancreatitis dataset were identified using the criteria of p-value < 0.05 and|log2 (fold change)| >1.0. Afterwards, the differential expression patterns of DEGs were presented using volcano plots and heatmaps using the ‘ggplot2’ and ‘pheatmap’ packages in the R software.

### Functional enrichment analysis

The Metascape (http://metascape.org) database allows for enrichment analysis using Gene Ontology to further understand the potential biological significance of the common genes in the DEGs. The hallmark gene set from the Molecular Signature Database (MSigDB) and the KEGG pathway were utilized for enrichment analysis. GSEA was used to analyze DEGs, and the standard was a P value < 0.05, using the “GSEABase” and “clusterProfiler” packages.

### Identification of candidate small molecules

cMAP (https://clue.io) [16] is a gene expression profile database based on gene expression signature interventions that reveal relationships between diseases, genes, and small molecule compounds. In this study, DEGs were included in the cMAP online database to identify potential acute pancreatitis treatments for small-molecule drugs. In the end, the top ten compounds with the highest enrichment scores were identified.

### Molecular docking verification

We conducted molecular docking validation between the small-molecule drugs predicted in cMAP and the potential target proteins associated with acute pancreatitis. The reliability of drug therapy for acute pancreatitis was assessed based on the binding energy magnitude. PubChem mol2 file formats were used to obtain the structures, and RCSB Protein Data Bank (PDB, http://www.rcsb.org/) for the crystallographic structures of the core targets. Initially, target proteins were dehydrated, ligands removed using PyMOL 2.3.0 software, and subsequently saved in PDB format. The processed target protein was then imported into AutoDock Tools 1.5.6 software for hydrogenation, charge calculation, and saved in PDBQT format. Mol2 files of small molecule drugs were imported into AutoDock Tools 1.5.6 software, where total charge was determined, charge was assigned, flexible rotatable bonds were identified, and saved in PDBQT format. Grid box data for the protein of interest was then obtained. Finally, Autodock Vina 1.1.2 was utilized for molecular docking, and the results were visualized using PyMOL 2.3.0 software.

### Machine learning

Two machine learning algorithms, the minimum absolute shrinking age and selection operator (LASSO) algorithm and the support vector machine recursive feature elimination (SVM-RFE) algorithm, were used in this study to screen for significant diagnostic or prognostic variables. LASSO was performed using the “glmnet” package in R and is a regression analysis algorithm that applies regularization for variable selection [11]. In this study, the LASSO algorithm effectively reduced the dimensionality from 1497 original features to 13 meaningful features. This reduction was achieved through the use of the LASSO algorithm with cross-validation, utilizing the mean squared error as the cost function. Subsequently, the algorithm identified and retained a subset of more important features based on the mean squared error (this process uses a penalty parameter determined by tenfold cross-validation to prevent overfitting in modeling). SVM-RFE is a commonly utilized supervised machine-learning technique for classification and regression tasks, aimed at identifying optimal variables through the removal of SVM-generated eigenvectors. The “e1071”, “kernlab”, and “caret” packages were employed for conducting SVM analysis. The “rfe” function was utilized for SVM analysis, with the minimum cross-error serving as the gene selection criterion and the “svmRadial” method chosen as the parameter [12]. The genes that overlap between the LASSO and SVM-RFE algorithms will be further analyzed.

### ROC of diagnostic biomarker

Continuous variables were compared using the nonparametric Kruskal-Wallis test. The degree of efficacy of each diagnostic biomarker was assessed using receiver operating characteristic (ROC) curves by R software package ‘pROC’, which is the gold standard to prove the diagnostic accuracy and test the efficacy of diagnostic biomarkers in the GSE3644 cohort.

### Immune infiltration analysis

Gene expression profiles (GEPs) from normalized AP genes were compared with the gene set using GSVA (R package). The ssGSEA algorithm classifies gene sets based on their common biological functions, chromosomal localization, and physiological regulation [13]. The gene sets consist of 782 genes that are utilized in the prediction of the abundance of 28 tumor-infiltrating immune cells (TIICs) within individual tissue samples. The enrichment of 28 TIICs in acute pancreatitis was demonstrated by comparing standardized gene expression profiles specific to the condition with a designated set of genes.

### Experimental groups and reagent treatments

Male wild-type C57BL/6 mice, aged 6 to 8 weeks, were obtained from Huachuang Sino Pharmaceutical Technology Co., located in Jiangsu, China, and were maintained under specific pathogen-free conditions. Animal housing and experimental procedures were conducted in a specific pathogen-free animal room and super-clean bench in the laboratory, respectively. All experimental protocols were approved by the Animal Care and Use Committee of Anhui Medical University (Ethics Committee Approval Number: LLSC20200404). The study followed the ARRIVE guidelines (https://www.nc3rs.org.uk/arrive-guidelines).

Acute pancreatitis model: The acute pancreatitis model was induced in a specific pathogen-free (SPF) animal room with slight modifications to the previously described protocol [14]. An intraperitoneal injection of 3.3 g/kg L-arginine (Sangon Biotech (Shanghai)) was used to induce l-arginine pancreatitis; 72 h after the first injection, the animals were sacrificed. Experimental groups and sample acquisition: T In order to investigate the effect of recombinant Pero orexin 4 (Prdx4) on acute pancreatitis, mice were randomly divided into three groups (6 mice each). Prdx4 group (Prdx4): mice were intraperitoneally injected with L-arginine diluent and then injected with Prdx4 via the tail vein 0.5 h later (rmPrdx4 2 mg/kg in 100 µL PBS). Acute pancreatitis group (AP) mice were intraperitoneally injected with L-arginine diluent and then injected with PBS equivalent to Prdx4 via the tail vein 0.5 h later. Control group (Con): Mice were intraperitoneally and intravenously injected with equal amounts of PBS with L-arginine and Prdx4 at corresponding times.

### Pancreatic histopathology and immunohistochemical staining

Hematoxylin and eosin were used to stain the sections of the embedded pancreas after they were embedded in paraffin. Histological scores of HE-stained sections were performed by two researchers in a double-blind setting with reference to previous studies. Edema, acinar necrosis, inflammatory infiltrate, and perivascular infiltrate were each scored using a 0- to 4-point scale. The severity of pancreatic injury was calculated as the sum of the scores as previously described [15].

Pancreas sections were deparaffinized and hydrated. The sections underwent antigen retrieval at a high temperature and were blocked for 1 h using Bovine Serum albumin (BSA). Using primary antibodies diluted 1:2000, the sections were incubated overnight at 4 °C after blocking. The primary antibodies used were against Prdx4 (Prdx4, Scrviccbio, RatmAb #113,672). After incubation, the sections were washed three times with PBS and incubated with a 1:200 dilution of biotinylated secondary antibody (horseradish peroxidase (HRP)-conjugated goat anti-rabbit, Scrviccbio, RatmAb #GB23303). The reaction products were incubated with diaminobenzidine (DAB, China) and then counterstained with hematoxylin. Positive areas were quantified with ImageJ. All images were captured under high-power magnification (× 200) using a light microscope.

### Single-cell transcriptome data processing and analysis

GSE244963 raw data were downloaded from the GEO database, and the dataset (3 control samples and 3 severe acute pancreatitis samples) was derived from the GPL25947 Illumina NovaSeq 6000 (Rattus norvegicus). In the process of single-cell transcriptome data processing, we carried out the normalization, scaling, and clustering of cells and identified 9 main cell types in R v4.2.2 using Seurat v4.3.0. The single-cell extraction standard was nFeature_RNA > 200, and percent.mt < 5% was performed to remove double and dead cells. We then normalized the filtered gene barcode matrix using the ‘NormalizeData’ function. The top 1500 highly variable genes were found using the ‘vst’ method via the ‘FindVariableFeatures’ function; the highly variable genes were previously centered and scaled using ‘ScaleData ‘. We then performed a principal component analysis (PCA) based on these 1500 highly variable genes and reduced dimensionality using the Harmony package to remove the batch effect; then, Seurat’s ‘FindNeighbors’ and the ‘FindClusters’ and' RunUMAP' functions were used to display dimensionally reduced clusters on a 2D map generated by UMAP.

### Statistical analysis

Statistical analyses were conducted utilizing R (version 4.2.2), Strawberry Perl software (64-bit), and GraphPad Prism version 9.5.1. Results were displayed as mean ± SD. Differences between the two groups were compared by unpaired Student′s t-test. *P* < 0.05 was regarded as statistical significance.

## Results

### Data processing

**Fig. 1:**
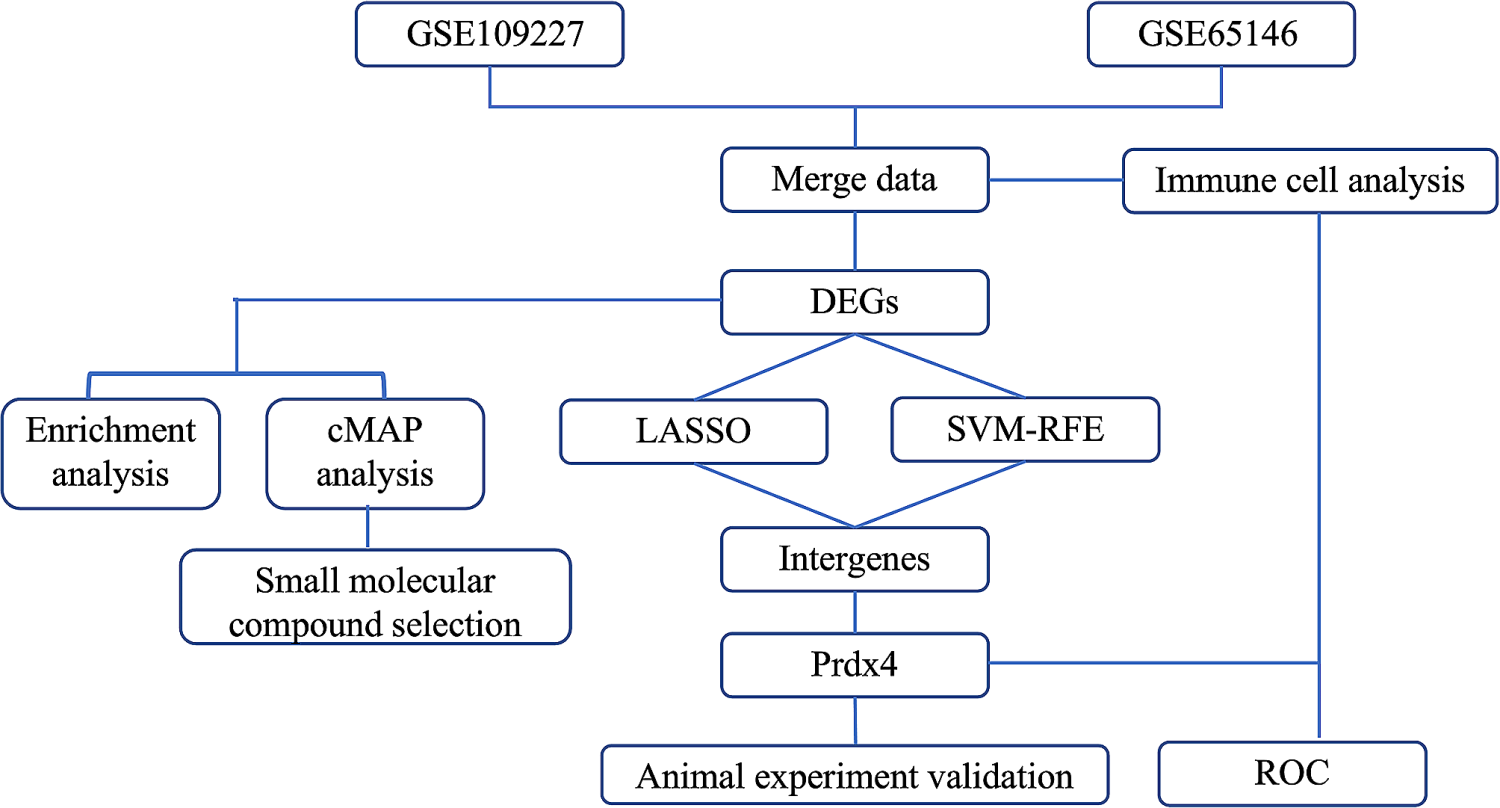
Flow chart of the study

**Fig. 2:**
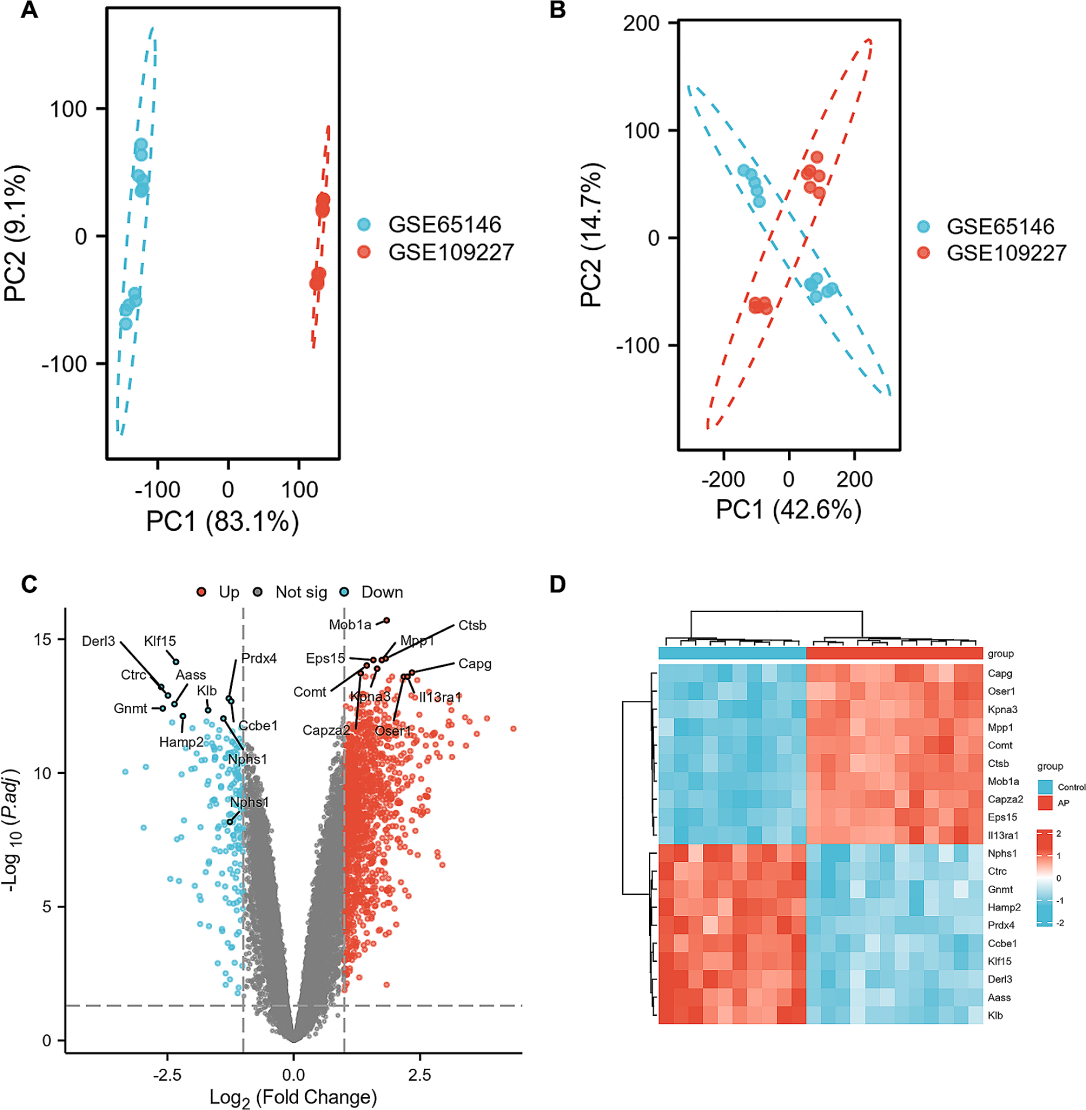
The integration of AP datasets and differential expression analysis of the integrated AP dataset. (**A**) PCA of three original AP datasets before batch-effect correction. (**B**) PCA of the integrated AP dataset after batch-effect correction. (**C**) The volcano plot representing AP DEGs in the integrated AP dataset. The upregulated genes are presented in red dots, whereas, the downregulated genes are presented in blue dots. (**D**) Heatmap plot of DEGs (up- and downregulated genes each displaying the top 10)

The bioinformatics analysis strategy is shown in Fig. 1. Two raw datasets of acute pancreatitis and control samples were collected from the GEO database and combined after carrying out batch effect removal. After batch correction, the integrated AP dataset was obtained and normalized, including 12 pancreatic tissue samples in the AP group and 12 control samples in the control group. As shown in Fig. 2A and B, the differences among two datasets were significantly decreased after batch effect removal.

### Identification of differentially expressed genes in acute pancreatitis

Differential analysis between combined AP and control samples revealed 1497 differentially expressed genes (DEGs) with the cut-off criterion of adjusted *p* < 0.05 and|log2 (fold change)| > 1, containing 1304 up regulated and 193 down regulated genes. Volcano plot and differential gene heatmap were applied to depict the expression pattern of DEGs (up- and down-regulated genes each displaying Top10) in the integrated AP dataset (Fig. 2 C and 2D).

### Functional analysis of potential genes

**Fig. 3:**
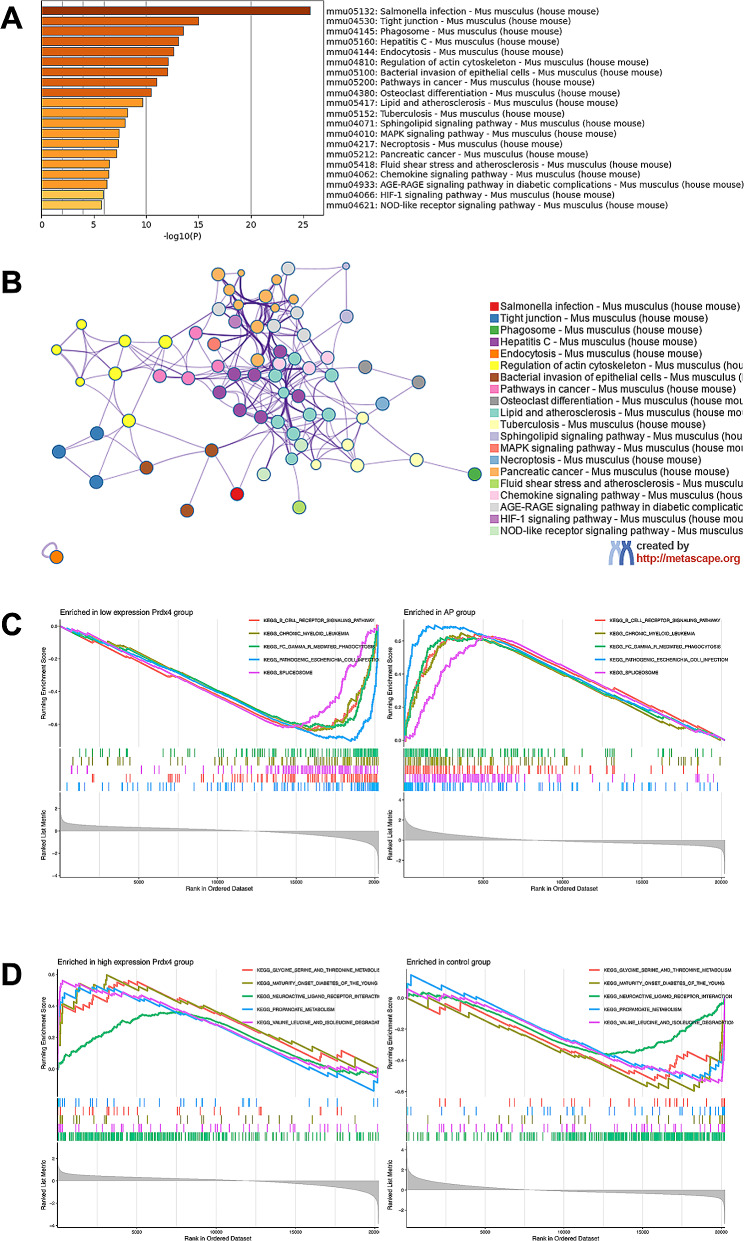
Functional enrichment analysis of DEGs. (**A**) Bar plot of DEGs functional enrichment terms. (**B**) Network relationship plots among all enriched terms. Colored by *p-*value, where terms containing more genes tend to have a more significant p-value. (**C**) GSEA enrichment analysis results in AP or low expression Prdx4 tissue samples. (**D**) GSEA enrichment analysis results in Control or high expression Prdx4 tissue samples

GO and pathway analysis with Metascape database to determine the biological function of potential genes. Potential genes were mainly involved in infection, tight junction, regulation of actin cytoskeleton (Fig. 3A). In addition, these potential genes are mainly enriched in leukocyte transendothelial migration, apoptosis and Fc gamma R-mediated phagocytosis. Figure 3B shows the relationships between the enriched terms. Table 1 lists the top 10 representative clusters of enrichment terms.

**Table 1:** Top 10 clusters with their representative enriched terms (one per cluster)

GO	Description	Count	%	Log10 (P)	Log10 (q)
mmu05132	Salmonella infection - Mus musculus (house mouse)	71	5.1	-26	-23
mmu04530	Tight junction - Mus musculus (house mouse)	44	3.2	-15	-13
mmu04145	Phagosome - Mus musculus (house mouse)	44	3.2	-14	-11
mmu05160	Hepatitis C - Mus musculus (house mouse)	41	3	-13	-11
mmu04144	Endocytosis - Mus musculus (house mouse)	54	3.9	-13	-11
mmu04810	Regulation of actin cytoskeleton - Mus musculus (house mouse)	48	3.5	-12	-10
mmu05100	Bacterial invasion of epithelial cells - Mus musculus (house mouse)	26	1.9	-12	-10
mmu05205	Proteoglycans in cancer - Mus musculus (house mouse)	44	3.2	-12	-10
mmu05135	Yersinia infection - Mus musculus (house mouse)	34	2.5	-11	-9.6
mmu05200	Pathways in cancer - Mus musculus (house mouse)	80	5.8	-11	-9.5

Moreover, GSEA results showed that B cell receptor signaling pathway, chronic myeloid leukemia, FC-gamma-R- mediated phagocytosis, pathogenic Escherichia collineation and spliceosome were mainly enriched in AP samples or low expression Prdx4 group (Fig. 3C). The glycine serine and threonine metabolism, maturity onset diabetes of the young, neuroactive ligand receptor interaction, propanoate metabolism, valine leucine and isoleucine degradation in control samples or high expression Prdx4 group (Fig. 3D).

### Identification of candidate small-molecular compounds for AP treatment

**Fig. 4:**
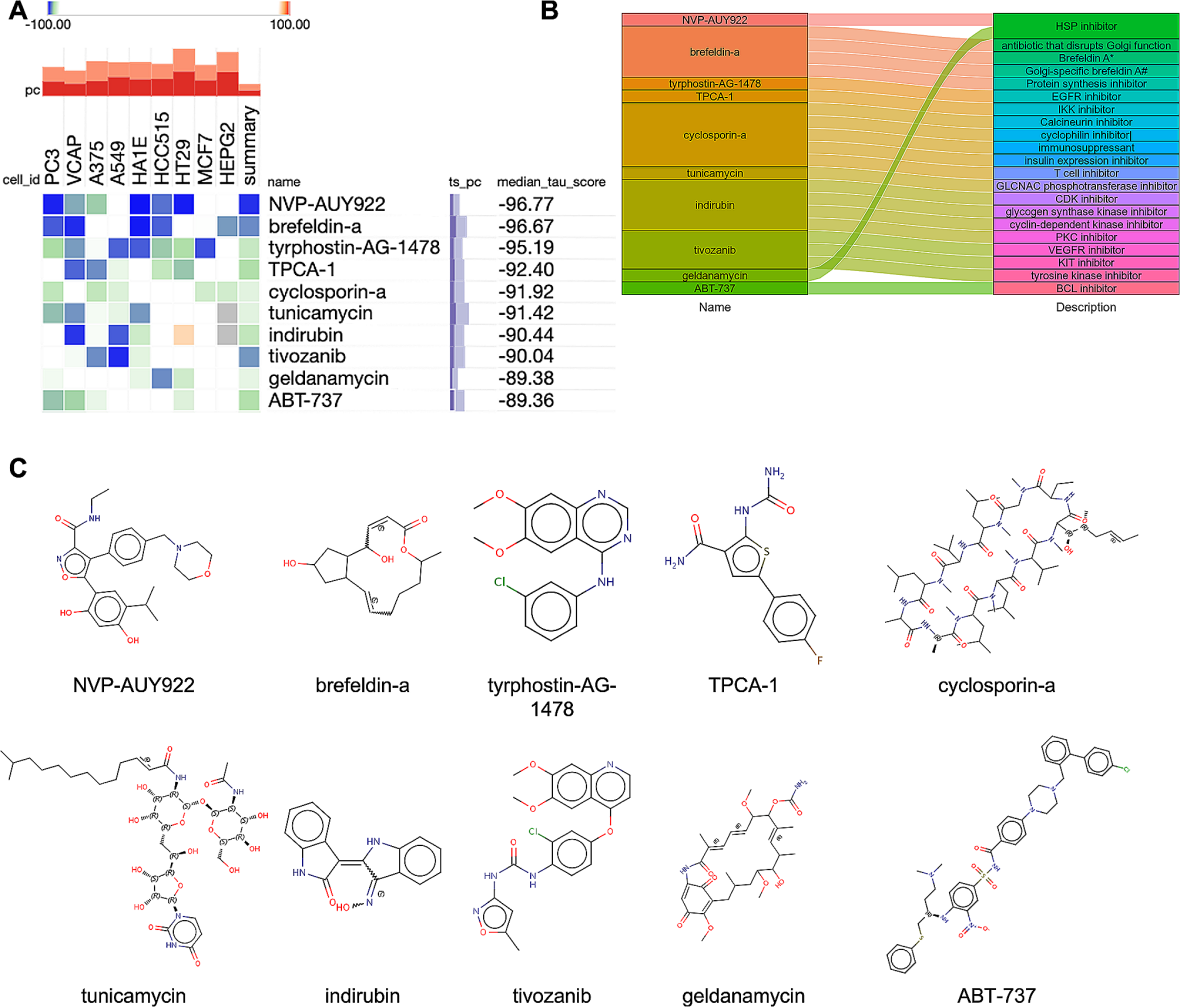
Screening of the potential small-molecular compounds for the treatment of AP via cMAP analysis. (**A**) The heatmap presenting the top10 compounds with the most significantly negative enrichment scores in 10 cell lines based on cMAP analysis, and descriptions of the top 10 compounds. (**B**) The chemical structures of those 10 compounds were shown. (**C**) cMAP connectivity map

To further investigate the potential small-molecular drugs that might exert a therapeutic effect in AP patients, the DEGs gene in AP samples of acute pancreatitis was imported into the connection map (cMAP) database to predict reversible AP small molecular compounds. Following the significant inquiry, the top10 compounds including NVP-AUY922, brefeldin-a, tyrphostin-AG-1478, TPCA-1, cyclosporin-a, tunicamycin, indirubin, tivozanib, geldanamycin, and ABT-737 with the highest negative scores were considered to be potential pharmacological therapeutic agents for the treatment of acute pancreatitis (Fig. 4A and B). The description of the targeted pathways and chemical structures of these 10 compounds were displayed in (Fig. 4C).

### Prdx4 gene was identified and verified as diagnostic biomarker by LASSO and SVM-RFE

**Fig. 5:**
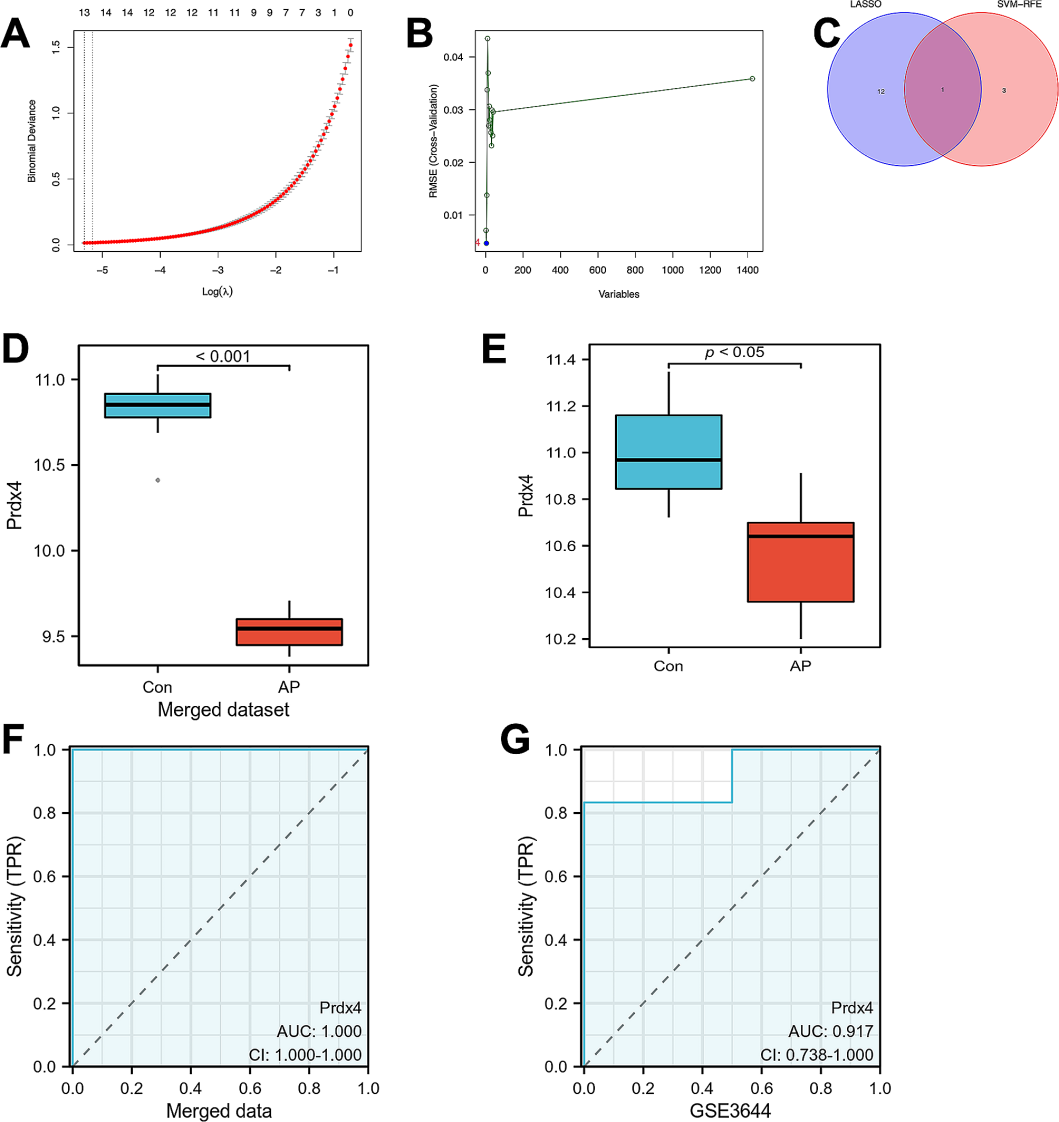
Screening of hub genes with diagnostic value via machine learning. (**A**) 13 diagnostic markers were screened by the least absolute shrinkage and selection operator (LASSO) logistic regression algorithm. (**B**) 4 diagnostic markers were screened by a support vector machine-recursive feature elimination (SVM-RFE) algorithm. (**C**) Venn diagram of variables intersecting LASSO and SVM-RFE algorithms. (**D**) The expression level of hub gene Prdx4 in the merged dataset between AP and control groups. (**E**) The expression level of hub gene Prdx4 in the in the validation cohort between AP and control groups. (**F**) Analyze of diagnostic validity of the diagnostic marker Prdx4. (**G**) ROC validation of diagnostic validity of the diagnostic marker Prdx4 in the validation cohort. AUC = 0.917.

Candidate diagnostic biomarkers were screened by two different algorithms. We utilized the least absolute shrinkage and selection operator (LASSO) logistic regression algorithm to identify 13 meaningful feature variables relative to acute pancreatitis from DEGs (Fig. 5A, Supplementary Table S1). The support vector machine-recursive feature elimination (SVM-RFE) algorithm was used to classify 16 features among whole DEGs (Supplementary Table S2) and identify a subset of 4 significant features (Fig. 5B, Supplementary Table S3). Thus, the only meaningful features that overlap are genes of the two algorithms are selected, as shown in Fig. 5C.

After obtaining the gene Prdx4, we first analyzed their expression levels in the merged dataset (Fig. 5D, ) which shows its significantly different expression level between the control group and the AP group. Then the GSE3644 dataset was used as a validation cohort to verify the accuracy of the above analysis results as well as the expression level of this candidate diagnostic biomarker. Compared with the control group, the expression level of Prdx4 in AP samples was significantly down-regulated (Fig. 5E). To further validate the diagnostic efficacy of Prdx4, we performed ROC validation using the merge data and the GSE3644 dataset, respectively, which is the gold standard for evaluating diagnostic accuracy and survival. The AUC for Prdx4 in the merged data queue is 1.000(Fig. 5F). In addition, the AUC of Prdx4 in the GSE3644 dataset was 0.917 (Fig. 5G), indicating that Prdx4 has definite diagnostic value.

### Molecular docking verification

Prdx4 is an important anti-inflammatory and antioxidant molecule, and its expression is decreased in AP group, which is more likely to be used as a therapeutic target [22, 23]. Using Autodock Vina 1.1.2 software, the screened small molecule drugs were docked the core target Prdx4. Studies have shown the lower the binding energy, the more stable the binding conformation and the greater the likelihood of action [24].

**Fig. 6:**
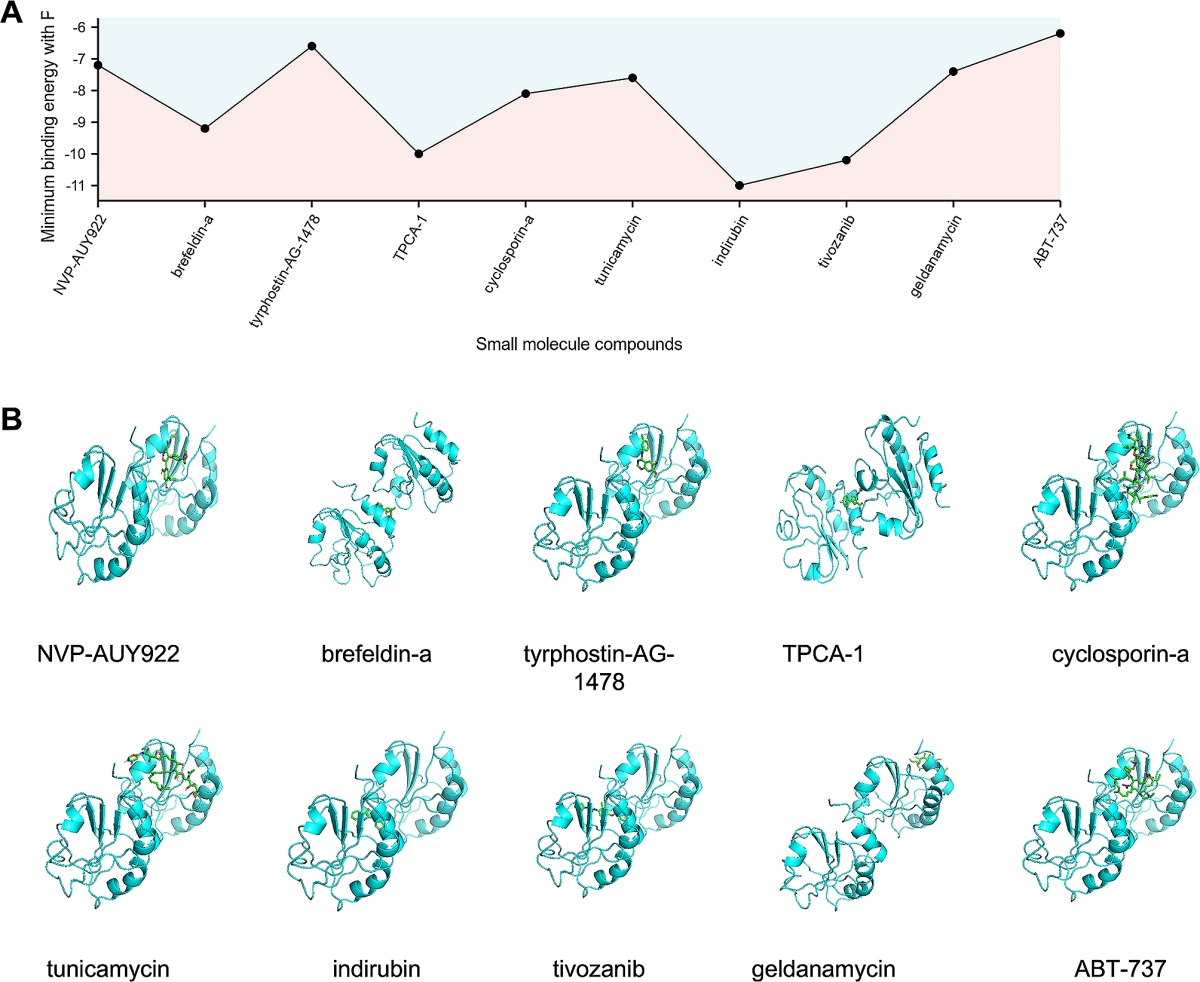
Docking diagram of small molecule drugs with targets. (**A**) Line diagram of the lowest binding energy for molecular docking. (**B**) Docking diagram of NVP-AUY922, brefeldin-a, tyrphostin-AG-1478, TPCA-1, cyclosporin-a, tunicamycin, indirubin, tivozanib, geldanamycin, and ABT-737 docked to Prdx4, respectively

As can be seen from Fig. 6A, the minimum binding energy between the ligand and the receptor is mostly less than − 7.0 kcal·mol^− 1^, indicating that the target protein has a good affinity with the active ingredient, and small molecule drugs are likely to act on the target Prdx4. Small molecule drug docking targets with the lowest binding energy were selected for docking visualization (Fig. 6B).

### Correlation analysis of hub genes with invading immune cells in AP

**Fig. 7:**
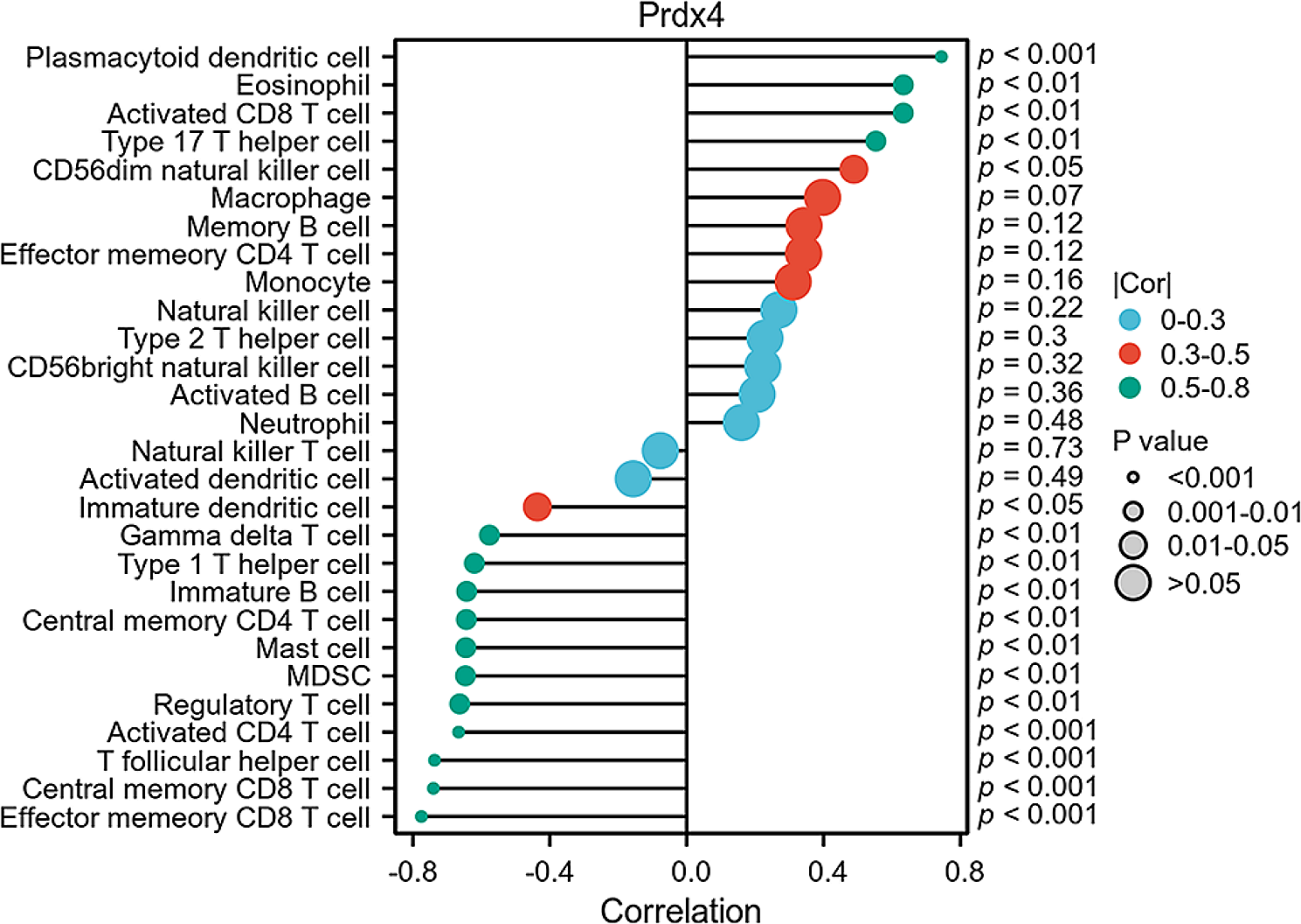
A diagram of the association between infiltrating immune cells and the pivot gene Prdx4

We found that the function and pathway analysis in AP show that they are closely related to inflammation and immune processes. ssGSEA was used to deduce the characteristics of immune cells and investigate the correlation between immune regulation and diagnostic biomarkers in AP. We investigated the relationship between the expression of the gene Prdx4 and the proportion of differential infiltrating immune cell types. As illustrated in Fig. 7, the central gene Prdx4 exhibited significant correlations with the aggregation of various immune cell types in acute pancreatitis. Specifically, Prdx4 demonstrated positive associations with Plasmacytoid dendritic cells, Eosinophils, Activated CD8T cells, Type 17 T helper cells, and CD56dim natural killer cells. Conversely, negative correlations were observed with Immature dendritic cells, Gamma delta T cells, Type 1 T helper cells, Immature B cells, Central memory CD4 T cells, Mast cells, MDSCs, Regulatory T cells, Activated CD4 T cells, T follicular helper cells, Central memory CD8 T cells, and Effector memory CD8 T cells.

### The validation of the expression pattern of hub genes Prdx4

**Fig. 8:**
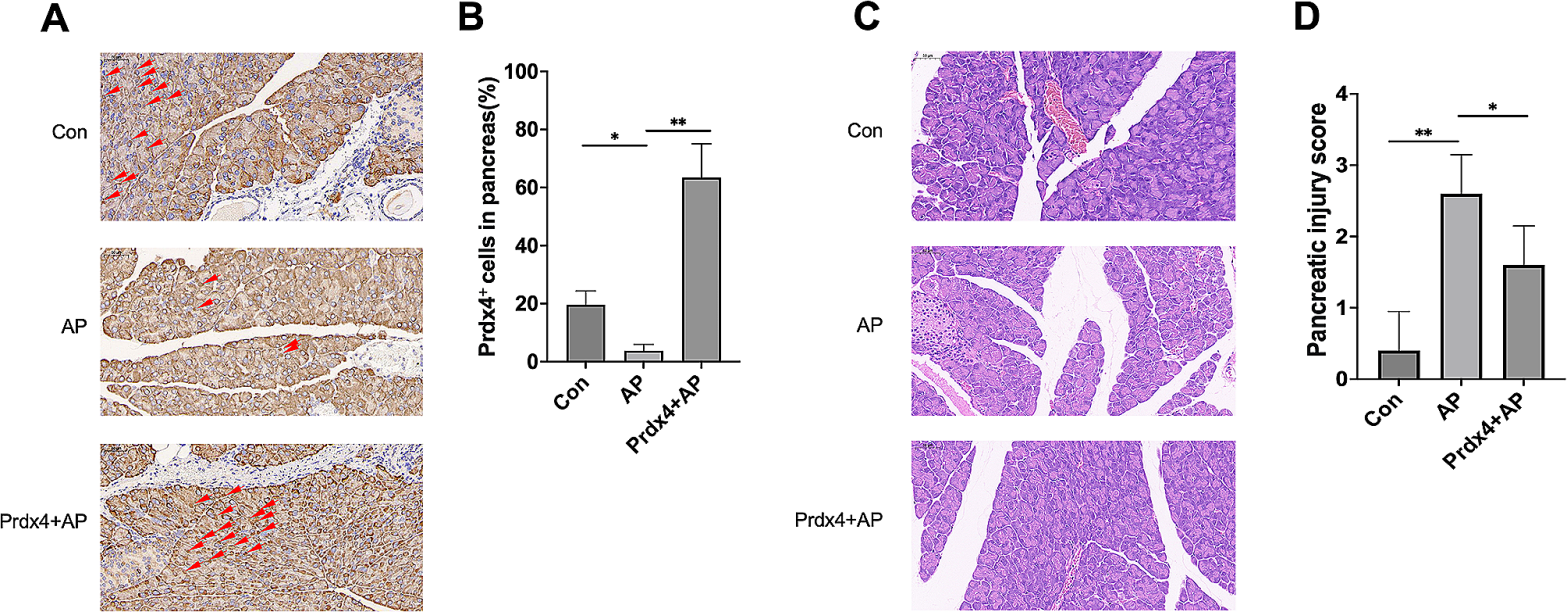
The hub gene Prdx4 plays a critical role in occurrence of AP. (**A**) The expression of Prdx4 in pancreatic tissue of AP model mice was detected by immunohistochemical staining (magnification, ×200). (**B**) Percentage of Prdx4^+^ cells in total number of cells in pancreatic tissue. (**C**) Representative histological sections of pancreatic tissue from AP model mice (hematoxylin and eosin staining; magnification, ×200)(**D**) The pathological pancreatic injury score based on histological sections

In order to further confirm the accuracy of the above comprehensive bioinformatics analysis, we first detected the expression of Prdx4 in the pancreatic tissues of the mouse model of pancreatitis. The results showed that the pancreatic injury score was significantly increased in the L-arginine-induced pancreatitis group. Meanwhile, immunohistochemistry showed that the expression of Prdx4 was significantly increased in the pancreatitis group Fig. 8A and B, suggesting that Prdx4 may be used as an auxiliary diagnostic marker for acute pancreatitis.

To further demonstrate that Prdx4 may be involved in the progression of AP and is expected to be a target for AP therapy, we administered recombinant Prdx4 to AP mice. The results showed that compared with the acute pancreatitis group, pancreatic edema, acinous necrosis, inflammatory infiltration and perivascular infiltration were significantly reduced in the treatment group, and the pancreatic injury score was decreased (Fig. 8C and D), suggesting that Prdx4 may be a therapeutic target for AP.

### The expression of Prdx4 was different in various cells during acute pancreatitis

**Fig. 9:**
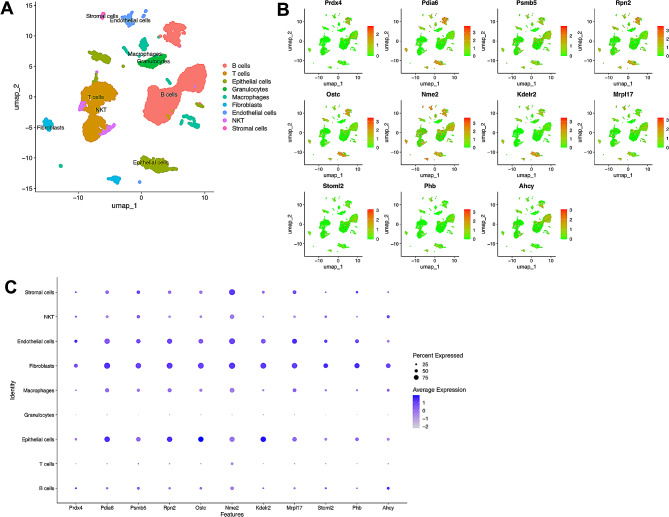
The expression of Prdx4 was different in various cells during acute pancreatitis. (**A**) UMAP plot of all the single cells, with each color-coded for the 9 major cell types. (**B**) UMAP plots showing the expression of Prdx4 and its co-expressed genes. (**C**) Bubble plot showing the expression levels of Prdx4 and its co-expressed genes. The size of each dot represents the percent expressed; average expression is shown by color

To examine the expression of Prdx4 in the external organs of the pancreas during acute pancreatitis, a single-cell sequencing analysis was conducted on ileum tissues from rats with acute pancreatitis sourced from GSE244963. The findings revealed highly expression of Prdx4 in fibroblasts, endothelial cells, and epithelial cells during acute pancreatitis, as well as in NKT cells, macrophages, granulocytes, and B cells (Fig. 9A and C). To verify the accuracy of Prdx4 expression, the co-expressed genes of Prdx4 (top 10), including Ostc, Pdia6, Psmb5, Rpn2, Nme2, Kdelr2, Mrpl17, Stoml2, Phb, Pdia6 and Ahcy, were identified from the Harmonizome 3.0 database (https://maayanlab.cloud/Harmonizome/) (Supplementary Table S4). The expression patterns of these genes in each cell type were found to be consistent with that of Prdx4 (Fig. 9A and C). However, Prdx4 was not identified as a highly variable gene in the single cell analysis of control rats, potentially due to its low expression levels. Tissue expression data from the Harmonizome 3.0 database indicated that Prdx4 exhibited high expression levels in pancreatic tissue cells, specifically in Plpha cells (10.7), beta cells (10.5), and pancreatic islets (10.7), whereas its expression in the small intestine outside the pancreas was comparatively lower (8.3) (Supplementary Fig S1).

## Discussion

Acute pancreatitis is a common exocrine inflammatory disease of the pancreas, which can cause multiple organ dysfunction and lead to pancreatic necrosis, permanent organ failure, and even death [3]. The global incidence of AP was 30 to 40 cases 100,000 population per year and still showed a continuous increase [16, 17]. Therefore, the exploration of AP-related pathogenic genes will undoubtedly provide a broader clinical picture for the diagnosis and treatment of AP. On this basis, we compared the genes of the control group and AP pancreatic tissue to find more candidate genes related to the development of AP and the changes of immune cells during AP, hoping to further explore the possible underlying mechanism of AP.

In this study, a comprehensive bioinformatics analysis method was used to explore the pathogenic genes of acute pancreatitis, and it was speculated that inflammation and infection, as well as signaling pathways such as “B cell receptor signaling pathway”, “Fc gamma receptor mediates phagocytosis”, “pathogenic Escherichia collineation” and “spliceosome” might be the potential mechanisms of AP pathogenesis. Through machine learning, the Prdx4 gene was obtained from the intersection of LASSO and SVM-RF genes. Then the efficacy of Prdx4 in AP diagnosis was evaluated by ROC curve. The AUC of Prdx4 was 1.000 in the combined data cohort and 0.917 in the GSE3644 cohort, indicating the potential of the Prdx4 gene as a biomarker to predict the occurrence of AP. We further found that pancreatic tissue Prdx4 expression was significantly reduced in AP mice, and pathological pancreatic injury improved after treatment with recombinant Prdx4.

Multiple immune cells are involved in the pathogenesis of acute pancreatitis. Early and rapid immune suppression occurs in AP patients, with the degree of suppression correlating with disease severity [18]. The results of GSEA suggest that the “B cell receptor signaling pathway” may be a potential mechanism in the development of AP. B cells show a positive correlation with inflammatory markers like C-reactive protein, aiding in the accurate diagnosis and assessment of therapeutic outcomes in AP [19]. The susceptibility of immature B cells to negative selection is believed to be a crucial factor in the maintenance of immunological self-tolerance [20]. In the AP group, there was a notable increase in the proportion of immature B cells and a significant decrease in memory B cells, suggesting a state of immunosuppression or negative immunoregulation. The findings of this study indicate a negative correlation between Prdx4 and immature B cells, as well as a positive correlation trend with memory B cells. The observed positive correlation between Prdx4 and immunoactive cells, specifically activated CD8 T cells and Th17 cells, implies that diminished levels of Prdx4 in the context of acute pancreatitis could potentially facilitate immune tolerance. Recent studies have demonstrated that excitatory immunity induced by toll-like receptor 3 ligand polyI: C can alleviate acute pancreatitis by inhibiting neutrophil chemotaxis and reactive oxygen species production [21]. Conversely, Prdx4 is negatively correlated with activated CD4 T cells, effector CD8 T cells, and Th1 cells, indicating that low expression of Prdx4 may enhance immune activation in these cell types. We analyzed single-cell sequencing and found that Prdx4 was expressed in both B cells and T cells. During pancreatitis, the coexistence of immune activation and immune tolerance in various cells influences the overall direction of the immune response.

Effective pharmacotherapy for the treatment of acute pancreatitis is still lacking, so there is an urgent need to explore potential drugs. Over the past few years, many important breakthroughs have been made in identifying small molecule compounds with therapeutic potential for a variety of diseases [22]. Small molecule compounds have the advantage of high tissue penetration, adjustable half-life, oral bioavailability, etc., which can produce better multiple therapeutic effects [23]. The compounds C42H60N4O6 and C28H29F3N4O5S can significantly improve AP damage in vitro and in vivo by inhibiting S100A9-vnn1 interaction, which has been confirmed in animal experiments, but clinical validation is still lacking [24]. Finding more potential small molecule compounds based on AP gene expression characteristics for targeted therapy through high-throughput screening will provide a broader choice for improving the prognosis of AP. The pathogenic genes associated with AP were analyzed by cMAP, and 10 small molecule compounds (NVP-AUY922, brefeldin-a et al.) were selected as candidate compounds. Notably, in the cMAP analysis, NVP-AUY922 showed the highest negative enrichment score, which means that it maximally reversed the expression of AP teratogenic genes. NVP-AUY922 is a potent inhibitor of heat shock protein 90, that interact with the Ca(2+)-activated K(+) (BK_Ca_) channel to largely increase Ca2^+^-activated K^+^ channels (*I*_K(Ca)_) in human pancreatic duct epithelial cells and restore pancreatic acinal cell function [25, 26]. Many studies have reported that NVP-AUY922 can alleviate Radiation-induced lung injury by inhibiting chaperone-mediated lysosomal degradation of GPX4 [27], and has anti-inflammatory and antioxidant effects on preventing sepsis-induced MODS [28]. Therefore, we speculate that the application of NVP-AUY922 may inhibit the occurrence and development of AP through a variety of mechanisms.

Gene Prdx4 was embarked from the intersection of genes between LASSO and SVM-RF. Then we assessed the efficacy of Prdx4 on AP diagnosis by ROC curves. The AUC of Prdx4 in the merge data cohort was 1.000 and in the GSE3644 cohort was 0.917 showing that gene Prdx4 has the potential to be a diagnostic biomarker. Studies have demonstrated that Prdx4 co-localizes with inflammasome components in extracellular vesicles from inflammasome-activated macrophages, and is a critical regulator of inflammasome activity, which can reduce the susceptibility of mice to LPS-induced septic shock [29]. In the study of Qingyi Granules ameliorating acute pancreatitis, it was found that Prdx4 may be involved in signaling during the pathological injury of severe acute pancreatitis by analyzing the proteomics of pancreatic tissue [30]. Findings in mice with aging-associated delayed wound healing show that aged hPrdx4^+/+^ mice exhibit attenuated oxidative stress and inflammation, decreased neutrophil counts, increased macrophage infiltration, increased angiogenesis, and elevated GF levels, whereas Prdx4 deficiency was associated with mortality in mice [31]. It is suggested that the decreased expression of Prdx4 may impair its important mechanisms of anti-inflammatory, antioxidant, and regulation of immune cell function, and may be a potential marker and key link in the pathogenesis of AP. We found that the pathological injury score of pancreatic tissue in mice with acute pancreatitis was significantly increased and accompanied by increased Prdx4 expression compared with the control group. After treatment with recombinant Prdx4, the pancreatic pathological injury of AP mice was significantly reduced. These results further strengthen the evidence that Prdx4 can be an important target for AP diagnosis and treatment.

## Conclusion

In summary, we identified a potential genetic marker Prdx4 associated with AP occurrence, which undoubtedly provide a molecular target for pharmaceutical design.

### Electronic supplementary material

Below is the link to the electronic supplementary material.

Supplementary Material 1

Supplementary Material 2

Supplementary Material 3

## Data Availability

Data supporting the results of this study are available from the corresponding author upon reasonable request.
