# Supplementary material for: Revealing Prdx4 as a potential diagnostic and therapeutic target for acute pancreatitis based on machine learning analysis
Source: BMC Med Genomics. 2024 Apr 19;17:93. doi: 10.1186/s12920-024-01854-2 (PMC11027343; doi:10.1186/s12920-024-01854-2)
Supplement: Supplementary file 1 — Supplementary Material 1 [file 12920_2024_1854_MOESM1_ESM.docx]

**Table S1: 13 features of LASSO**

| Feature | Coefficients |
| --- | --- |
| Mob1a | 1.56795833 |
| Mpp1 | 1.01459986 |
| Eps15 | 0.04645194 |
| Capza2 | 0.78655619 |
| Myo5a | 0.09870838 |
| Arpc3 | 0.59075278 |
| Ctrc | -0.4096909 |
| Cdc42se2 | 0.1203677 |
| Prdx4 | -0.8124232 |
| Ccbe1 | -0.318117 |
| Ssu72 | 0.15986789 |
| Padi2 | -0.851835 |

**Table S2: 16 features of SVM-RFE**

| Points | Variables | RMSE | Rsquared | MAE |
| --- | --- | --- | --- | --- |
| 1 | 2 | 0.007071 | 1 | 0.009333 |
| 2 | 4 | 0.004641 | 1 | 0.00618 |
| 3 | 6 | 0.013762 | 0.999993 | 0.025471 |
| 4 | 8 | 0.033777 | 0.997726 | 0.023157 |
| 5 | 10 | 0.043496 | 0.998823 | 0.036286 |
| 6 | 13 | 0.036965 | 0.998823 | 0.034049 |
| 7 | 16 | 0.026938 | 0.99914 | 0.025463 |
| 8 | 19 | 0.030645 | 0.999361 | 0.024595 |
| 9 | 22 | 0.028024 | 0.999366 | 0.021674 |
| 10 | 25 | 0.026686 | 0.999006 | 0.019391 |
| 11 | 28 | 0.025671 | 0.998817 | 0.017399 |
| 12 | 31 | 0.023149 | 0.999599 | 0.016736 |
| 13 | 34 | 0.029918 | 0.999222 | 0.022965 |
| 14 | 37 | 0.025058 | 0.999511 | 0.02352 |
| 15 | 40 | 0.029553 | 0.999387 | 0.026113 |
| 16 | 1425 | 0.03587 | 0.999966 | 0.01292 |

**Table S3: 4 meaningful features of SVM-RFE**

| Gene ID | logFC | AveExpr | t | P.Value | adj.P.Val |
| --- | --- | --- | --- | --- | --- |
| Prdx4 | -1.285031 | 10.123357 | -19.076513 | 2.74E-16 | 1.64E-13 |
| Sec11c | -1.28283 | 10.052419 | -14.052178 | 2.79E-13 | 1.93E-11 |
| Ppp1r1b | -1.207082 | 7.729996 | -13.896434 | 3.57E-13 | 2.33E-11 |
| Prr23a1 | -1.305666 | 9.049037 | -12.722318 | 2.44E-12 | 1.02E-10 |
