# Supplementary material for: Revealing Prdx4 as a potential diagnostic and therapeutic target for acute pancreatitis based on machine learning analysis
Source: BMC Med Genomics. 2024 Apr 19;17:93. doi: 10.1186/s12920-024-01854-2 (PMC11027343; doi:10.1186/s12920-024-01854-2)
Supplement: Supplementary file 2 — Supplementary Material 2 [file 12920_2024_1854_MOESM2_ESM.docx]

**Table S4: Prdx4 Co-expressed genes (Top 10)**

| Rank | Gene Symbol | Pearson Correlation |
| --- | --- | --- |
| 1 | [PDIA6](https://maayanlab.cloud/archs4/gene/PDIA6) | 0.7658263444900513 |
| 2 | [PSMB5](https://maayanlab.cloud/archs4/gene/PSMB5) | 0.7433418035507202 |
| 3 | [RPN2](https://maayanlab.cloud/archs4/gene/RPN2) | 0.7241458296775818 |
| 4 | [OSTC](https://maayanlab.cloud/archs4/gene/OSTC) | 0.7194944024085999 |
| 5 | [NME2](https://maayanlab.cloud/archs4/gene/NME2) | 0.7111063003540039 |
| 6 | [KDELR2](https://maayanlab.cloud/archs4/gene/KDELR2) | 0.7057827711105347 |
| 7 | [MRPL17](https://maayanlab.cloud/archs4/gene/MRPL17) | 0.704841136932373 |
| 8 | [STOML2](https://maayanlab.cloud/archs4/gene/STOML2) | 0.7014265060424805 |
| 9 | [PHB](https://maayanlab.cloud/archs4/gene/PHB) | 0.7000718712806702 |
| 10 | [AHCY](https://maayanlab.cloud/archs4/gene/AHCY) | 0.6927328109741211 |
