# Supplementary figures and images for: Revealing Prdx4 as a potential diagnostic and therapeutic target for acute pancreatitis based on machine learning analysis
Source: BMC Med Genomics. 2024 Apr 19;17:93. doi: 10.1186/s12920-024-01854-2 (PMC11027343; doi:10.1186/s12920-024-01854-2)

Figure S1: Expression levels of Prdx4 across tissues


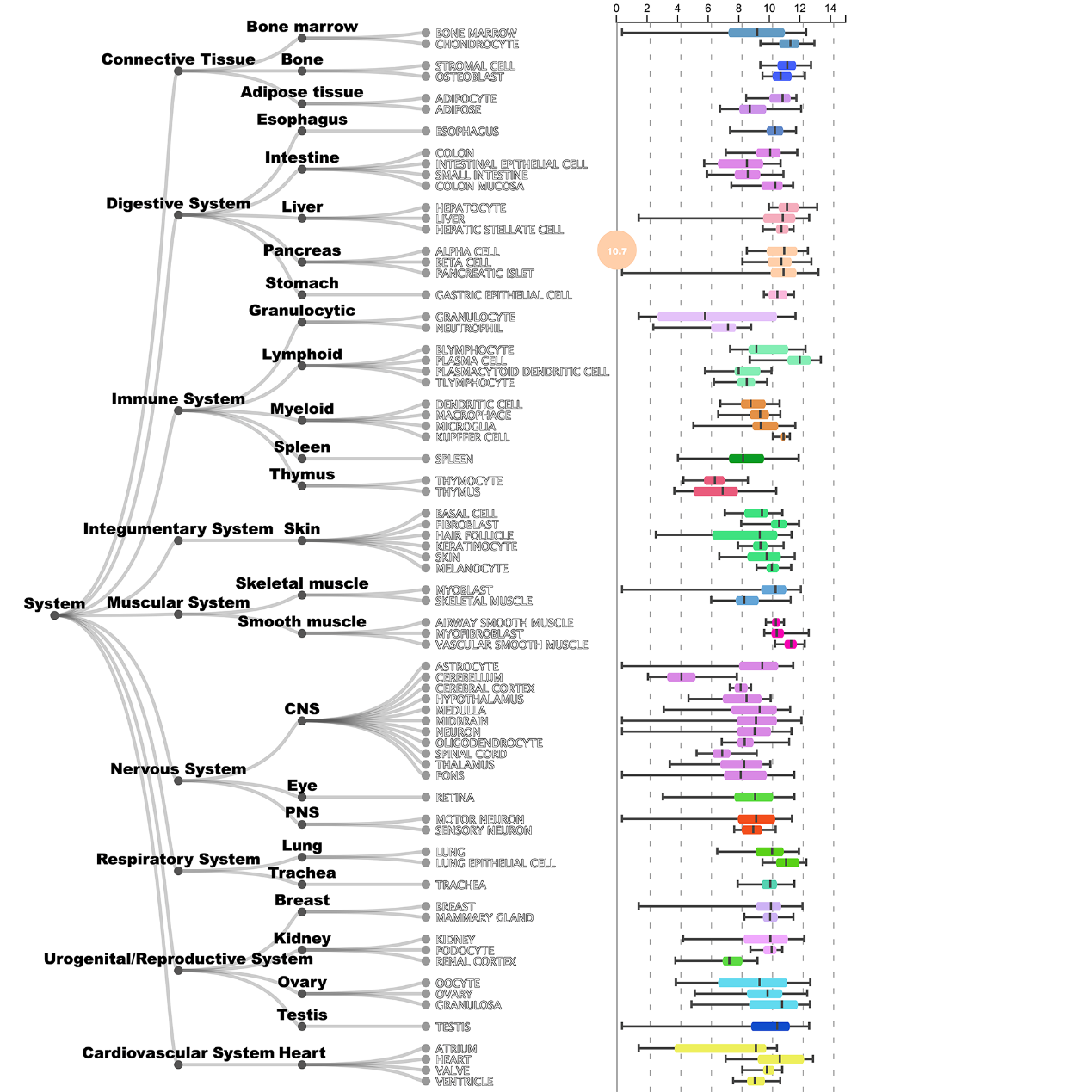

Supplement: Supplementary file 3 — Supplementary Material 3 [file 12920_2024_1854_MOESM3_ESM.docx]
